# Rapid growth of a long-standing dermal nodule in a young Black man: Clinical correlation in an ALK-rearranged epithelioid/spindle cell tumor

**DOI:** 10.1016/j.jdcr.2026.05.024

**Published:** 2026-05-15

**Authors:** Jungsoo Chang, Joseph S. Durgin, Michael J. Davis, Kimberly F. Breglio, Elisabeth A. Pedersen

**Affiliations:** a Department of Dermatology, University of Michigan, Ann Arbor, Michigan; b Division of Dermatopathology, Department of Pathology, University of Michigan, Ann Arbor, Michigan; c Division of Cutaneous Surgery and Oncology, Department of Dermatology, University of Michigan, Ann Arbor, Michigan

**Keywords:** ALK-rearranged neoplasm, epithelioid fibrous histiocytoma (EFH), PPFIBP1::ALK fusion, superficial ALK-rearranged myxoid spindle cell neoplasm (SAMS)

## Introduction

Advances in molecular studies and sequencing are increasingly resulting in reclassification of cutaneous neoplasms based on genetic features.^1^ However, for practicing dermatologists, pathology reports that incorporate extensive molecular terminology can be difficult to interpret, particularly when established entities are reclassified based on genetic alterations. One group of such entities is the emerging spectrum of anaplastic lymphoma kinase (ALK)-rearranged cutaneous and soft tissue tumors, which includes superficial ALK-rearranged myxoid spindle cell neoplasm (SAMS), epithelioid fibrous histiocytoma (EFH), and inflammatory myofibroblastic tumor.^2^ While molecular and histopathologic characterization of these entities is growing and has identified distinct immunophenotypic and morphologic profiles, clinical photographs and reported examples of management approaches remain limited.

Herein, we report a case of an ALK-rearranged cutaneous epithelioid/spindle cell neoplasm in a young Black man with a longstanding solitary dermal nodule that underwent rapid growth. We describe the integration of clinical, molecular, and immunophenotypic data in this case in relation to the current concepts in ALK-rearranged cutaneous tumors.

## Case report

**Fig 1:**
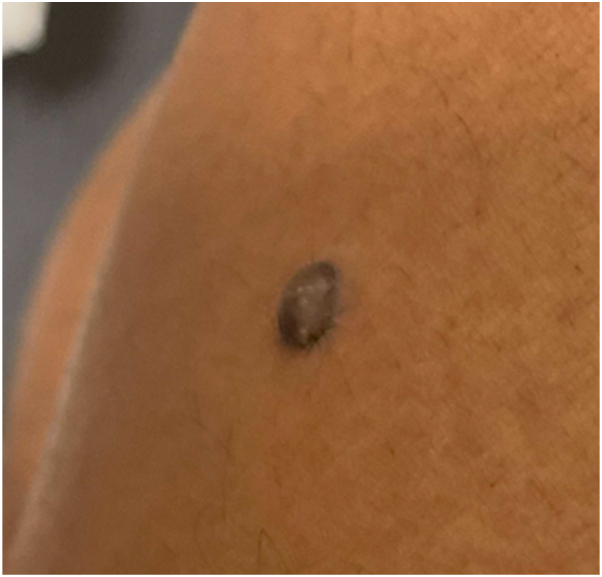
Clinical photograph of the brown nodule with a hyperpigmented ring and white punctate areas.

A 24-year-old Black male presented to an outside dermatology clinic with an asymptomatic lesion on his right anterior thigh that had been stable for several years but began to enlarge over the past 2 years. Physical examination demonstrated a 1.2 cm brown nodule on the right anterior thigh with a hyperpigmented peripheral ring and scattered white punctate regions (Fig 1). Given the rapid growth, a shave biopsy was performed. The differential diagnosis included dermatofibroma, an atypical melanocytic lesion, or an adnexal tumor.

**Fig 2:**
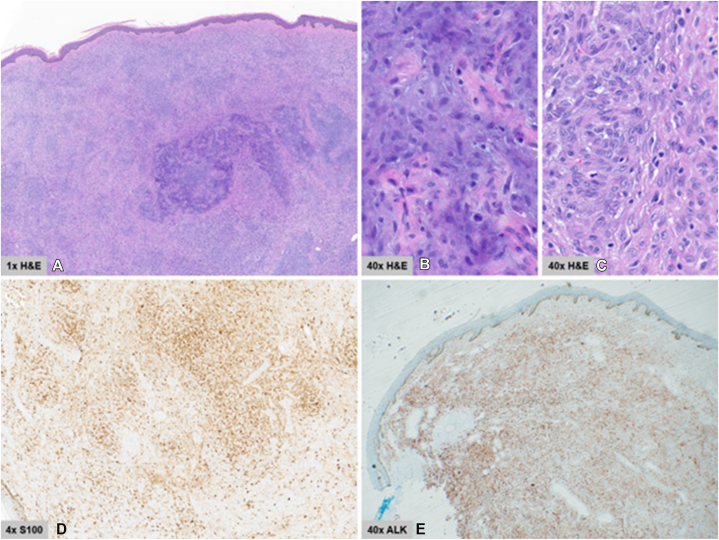
Histopathology of the tumor with **(A-C)** hematoxylin-eosin staining showing dermal proliferation of epithelioid to spindled cells with sheet-like to nested growth in a background of myxoid to collagenous stroma. A biphasic pattern is evident with more cellular areas containing ovoid to epithelioid cells. Immunohistochemistry staining with S100 **(D)** and ALK **(E)** positivity. *ALK*, Anaplastic lymphoma kinase.

Microscopic examination revealed a dermal proliferation of epithelioid to spindled cells growing in sheets and loose nests within a myxoid to collagenous stroma (Fig 2). Cytologically, the cells demonstrated eosinophilic cytoplasm and oval to crescentic nuclei with inconspicuous nucleoli. Immunohistochemical stains demonstrated positivity for S100, NKI/C3, and ALK, patchy expression of CD10 and patchy reactivity for CD34. Staining for EMA, ERG, MART-1, MiTF, p63, pancytokeratin, SMA, and SOX10 were negative in the proliferation (Fig 2). Factor XIIIA was not performed. Whole transcriptome RNA sequencing was performed for molecular characterization, identifying a pathogenic PPFIBP1::ALK fusion with no copy number changes. Interpreted in conjunction with the morphologic and immunophenotypic findings, the findings were most compatible with an (ALK)-rearranged cutaneous spindle and epithelioid cell tumor, on the continuum between SAMS and EFH.^3^ The lesion was excised with a narrow margin to ensure complete removal, and final pathology demonstrated no evidence of residual neoplasm. Limited clinical follow-up of 5 m demonstrated no clinical recurrence.

## Discussion

We present a case and clinical image of an ALK-rearranged epithelioid/spindle cell tumor spectrum harboring a rare PPFIBP1::ALK fusion in a patient with skin of color. Within the spectrum of this family of tumors, this lesion had the most resemblance to a SAMS, with features including a whorled growth pattern, variably myxoid stroma, S100 positivity, a lack of EMA expression, and a biphasic appearance with whorled spindled cells alongside more cellular areas with plump ovoid cells. However, all cases of SAMS previously reported had diffuse CD34 positivity (vs weak patchy positivity seen here), and the PPFIBP1::ALK fusion has been reported in EFH and inflammatory myofibroblastic tumor but not SAMS.^3^, ^4^, ^5^, ^6^, ^7^ Moreover, EFH typically presents as a well-circumscribed dermal nodule, often with an epidermal collarette, lacking the myxoid spindled cell whorls and concentric nodules that are characteristic of SAMS. Therefore, despite the weak CD34 and the finding of a novel fusion partner, this tumor is most compatible with SAMS, although with an unusual phenotype that may reflect an intermediate phenotype on the continuum with EFH.^5^, ^6^, ^7^

Multiple ALK fusion partners in superficial mesenchymal tumors have been identified with increased utilization of RNA sequencing; however, the relationship between clinical presentation, molecular fusion subtype, and natural history remains limited. PPFIBP1 is a rare fusion partner, and it encodes liprin-β1, a scaffolding adaptor involved in cell adhesion, migration, and synaptic organization.^3^^,^^8^^,^^9^ Although other ALK fusions are known to activate canonical oncogenic pathways such as RAS/MAPK and JAK/STAT,^10^ the specific downstream signaling associated with PPFIBP1::ALK in cutaneous neoplasms remains undefined.^3^^,^^8^

Patients with ALK-rearranged cutaneous tumors generally do well. However, the novelty of rare ALK fusion partners and limited clinical characterization may obscure the biologic potential of the tumor and, therefore, may complicate management decisions for both clinicians and patients alike. Given the uncertain biologic behavior of the rare PPFIBP1::ALK fusion, overlapping morphologic features that encompass both epithelioid and spindle cell tumor type, and reports of recurrence in ALK-rearranged tumors, complete surgical excision was performed, consistent with standard management.

While clinical images of morphologically similar tumors such as benign fibrous histiocytomas are widely available, published clinical images of these ALK-fusion tumors are rare. Notably, images of any ALK-rearranged cutaneous tumors have not been published in patients with skin of color.^4^, ^5^, ^6^ As ALK fusion-driven tumors are further characterized, images in diverse skin types will help both clinicians and pathologists understand the clinical spectrum of these tumors. By integrating clinical images with molecular characterization of a rare ALK fusion, this case broadens the clinicopathologic framework for these rare tumors and addresses a gap in representation of diverse presentations within the dermatologic literature.

## Conflicts of interest

None disclosed.
